# Electronic cigarette exposure on insulin sensitivity of ApoE gene knockout mice

**DOI:** 10.18332/tid/125399

**Published:** 2020-08-06

**Authors:** Kai Lan, Guangwei Zhang, Lijuan Liu, Ziwei Guo, Xianyu Luo, Hua Guan, Qi Yu, Enqi Liu

**Affiliations:** 1 Department of Clinical Medicine, Xi’an Medical University, Xi’an, China; 2 Department of Basic Medical Science, Xi’an Medical University, Xi’an, China; 3 School of Public Health, Xi’an Medical University, Xi’an, China; 4 Institute of Basic and Translational Medicine, Xi’an Medical University, Xi’an, China; 5 Laboratory Animal Center, Xi’an Jiaotong University Health Science Center, Xi’an Jiaotong University, Xi’an, China

**Keywords:** electronic cigarette, smoke exposure, diabetes, inflammatory injury, insulin sensibility

## Abstract

**INTRODUCTION:**

The current study aimed to investigate the effects of electronic cigarettes on insulin sensibility in ApoE gene knockout mice.

**METHODS:**

In total, 48 male ApoE gene knockout mice were randomly divided into four exposure groups: 1) electronic cigarette (e-cigarette) containing 12 mg/mL of nicotine, 2) e-cigarette without nicotine (0mg), 3) traditional cigarette (cigarette), and 4) fresh air (control). The first three groups were exposed to the associated smoke for 18 weeks. The body weight was recorded regularly in the four groups. After the last exposure, the concentrations of lipids, hs-CRP and TNF-α in serum were detected and the effect of electronic cigarettes on insulin tolerance was measured.

**RESULTS:**

The levels of serum lipid, hs-CRP and TNF-α in the e-cigarette, 0mg and cigarette groups were significantly increased compared with those in the control group (p<0.05). Also, the insulin tolerance in the e-cigarette, 0mg and cigarette groups was significantly decreased compared to that in the control group (p<0.05).

**CONCLUSIONS:**

Electronic cigarettes showed comparable effects to traditional cigarettes in influencing the metabolic functions in ApoE gene knockout mice.

## INTRODUCTION

The change of insulin sensibility is an important pathogenesis of type 2 diabetes. Although a number of studies have shown that cigarette smoking is closely related to metabolic diseases such as diabetes, it still needs further investigation to ascertain if smoking affects insulin sensibility^1-3^. With the emergence of electronical cigarettes (e-cigarettes) on the market as a substitute for traditional cigarettes, an increasing large population has become consumers of e-cigarettes^4,5^. Therefore, it is of great importance to fully understand the potential hazards of e-cigarettes. Nevertheless, the information about potential hazards is insufficient, especially the effects on body metabolism. Many studies have indicated that the levels of blood lipid and chronic inflammatory indexes are closely related to insulin sensibility. ApoE gene knockout mice are frequently used to investigate cardiovascular diseases; it is an ideal animal model to study the diseases related to the changes of blood lipids^6^. Therefore, the aim of the current study is to explore the effect of e-cigarette exposure on insulin sensibility based on the determination of serum lipids and chronic inflammatory indexes in order to clarify the potential hazards of e-cigarettes.

## METHODS

### Animals and smoking exposure

Forty-eight male ApoE knockout mice aged 6 weeks were purchased from the Experimental Animal Center of Xi’an Jiaotong University. According to the suggested formula containing 0.15% of cholesterol and 21% of fat (Wako, Japan), the diet used in the current study was prepared and supplied by Beijing Keao Xieli Feed Co, Ltd (Beijing, China). All the mice were randomly divided into four exposure groups: 1) e-cigarettes containing 12 mg/mL of nicotine (e-cigarette), 2) electronic cigarettes without nicotine (0 mg), 3) traditional cigarettes (cigarette), and 4) fresh air (control). We used a bionic simulation of human respiratory system smoking generating device (provided by the Central Laboratory of the Public Health College of Xi’an Medical College). A gas smoke generating device was docked with the intake port to ensure an airtight seal. The balloon device simulated the human respiratory system in its ability to absorb target gas, and the smoke was discharged from the outlet to the intake port. The e-cigarette group was given commercial electronic cigarettes with a 12 mg/mL nicotine content, while the 0mg group was given the same type of e-cigarette but without the nicotine content. The cigarette group was given ordinary commercial filter cigarettes with 12 mg tar and 0.8 mg nicotine content. The control group was given fresh air. Smoking exposure was conducted three times a day, 30 min each time, for a period of 18 weeks. Body weights were recorded once every two weeks. During the assay of blood glucose, the blood samples were collected from the tail vein. At the end of the experiment, the mice were sacrificed followed by collection of blood via cardiac puncture after anaesthesia. Blood was centrifuged at 2000 rpm at 4°C for 20 min and serum samples were stored at -80°C. All animal experiments were performed in accordance with the guidelines of the Xi’an Medical University (Shaanxi, China), and were approved by the Ethics Committee of Xi’an Medical University (XYLS2018061). The present study was performed in accordance with the recommendations of the Council for International Organization of Medical Sciences. The datasets used and analyzed during the current study are available from the corresponding author upon reasonable request.

### Detection of blood lipids and inflammatory indexes

The levels of total cholesterol, triglyceride, low-density lipoprotein and high-density lipoprotein were determined by automatic biochemical analyzer. Hypersensitive C-reactive protein and serum tumor necrosis factor-alpha were determined by ELISA (Wuhan Huamei Bioengineering Co., Ltd, Wuhan, China) according to the instructions.

### Effect of e-cigarette on insulin tolerance

The insulin dosage in the insulin tolerance test was 0.7 U/kg Insulin diluted with normal saline at a concentration of 0.7 U/mL. Blood glucose was measured at 0, 15, 30, 60, 90, 120 and 150 min after insulin injection in each group. Using Graph Pad Prism 6.0 software, taking time as abscissa and the blood glucose value measured at each time point as ordinate, a blood glucose curve was generated and the area under the curve calculated.

### Statistical analysis

Data were expressed as mean ± SD. Differences between groups were evaluated by analysis of variance (ANOVA) with an unpaired t-test. Graph Pad Prism 6.0 was used for statistical analysis. A p<0.05 was considered to be statistically significant.

## RESULTS

### Effect of e-cigarette on body weight

**Figure 1:**
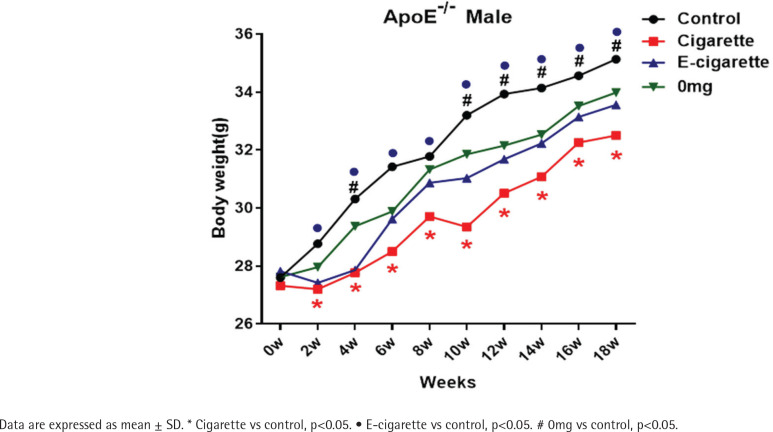
Body weight changes of ApoE-/- mice during smoke exposure for 18 weeks (n=12)

After 18 weeks of exposure, changes in the body weight of mice in the e-cigarette, 0mg, and cigarette groups, were lower than in the control group, and differences were statistically significant (p<0.05; Figure 1).

### Effect of e-cigarette on blood lipids

After 18 weeks of smoking exposure, the concentrations of blood lipids in the exposure groups are shown in Table 1. The levels of total cholesterol, triglyceride and low-density lipoprotein were significantly increased in the e-cigarette, 0mg and cigarette groups, compared to the control group (p<0.05). Also, The HDL levels in the exposure groups were lower compared to the control group and differences were statistically significant (p<0.05).

**Table 1:** Concentrations (mg/dL) of blood lipids in ApoE-/- mice in four tested groups after 18 weeks of smoke exposure (n=12)

*Group*	*TC*	*TG*	*LDL-C*	*HDL-C*
E-cigarette	749.31±29.78^a^^,^^b^	623.71±24.12^a^^,^^b^	453.59±15.32^a^	26.47±2.66^a^
0mg	731.97±28.17^a^^,^^b^	614.68±22.89^a^^,^^b^	451.56±15.61^a^	27.07±2.75^a^
Cigarette	853.13±37.93^a^	705.34±26.93^a^	462.63±15.83^a^	25.56±2.87^a^
Control	614.72±21.87^b^	418.57±14.11^b^	417.39±13.27^b^	39.62±3.12^b^

TC: total cholesterol; TG: triglyceride; LDL-C: low-density lipoprotein cholesterol; HDL-C: high-density lipoprotein cholesterol.

a Compared with the control group, p<0.05.

b Compared with the cigarette group, p<0.05.

### Effect of e-cigarette on inflammatory indexes

After 18 weeks of exposure, the concentrations of hs-CRP and TNF-α in the exposure groups are summarized in Table 2. It was found that the traditional cigarette, the electronic cigarette with 12 mg/mL of nicotine and without nicotine, significantly elevated the levels of hs-CRP and TNF-α (p<0.05).

**Table 2:** Concentrations (ng/mL) of serum hs-CRP and TNF-α in ApoE-/- mice in four tested groups after 18 weeks of smoke exposure (n=12)

*Group*	*hs-CRP*	*TNF-α*
E-cigarette	4.74±0.59^a^	2.14±0.27^a^^,^^b^
0mg group	4.65±0.57^a^	1.95±0.25^a^^,^^b^
Cigarette	4.98±0.61^a^	2.83±0.31^a^
Control	3.21±0.39^b^	1.73±0.23^b^

a Compared with the control group, p<0.05.

b Compared with the cigarette group, p<0.05.

### Effect of e-cigarette on insulin tolerance

**Figure 2:**
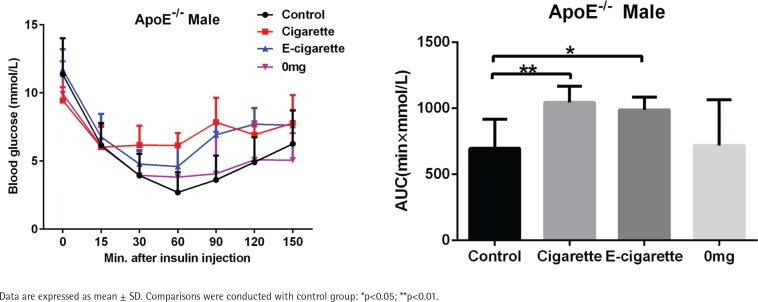
Effects of cigarette exposure on the insulin tolerance of ApoE-/- mice in four tested groups (n=12)

The concentrations of blood glucose in the exposure groups are plotted versus time in Figure 2 (left). For the control group versus cigarette group, the blood glucose number was statistically different at 60 and 90 min after insulin injection, while for the control group versus e-cigarette group, the blood glucose number was statistically different at 90 and 120 min after insulin injection. The decline in the blood glucose curve in the cigarette group and the e-cigarette group was significantly slowed down. The areas under the curve (AUCs) were calculated and are plotted in Figure 2 (right). It was found that e-cigarette with 12 mg/mL nicotine and traditional cigarette significantly enhanced the AUCs, whereas e-cigarette without nicotine did not increase the AUC compared to the control group, indicating that insulin tolerance was significantly decreased by the e-cigarette with 12 mg/mL nicotine and by the traditional cigarette.

## DISCUSSION

A decrease in insulin sensitivity is the initial pathology of type 2 diabetes. It is found that the enlarged adipocytes attract macrophages and secrete inflammatory molecules, while the glucose utilization in the body is affected by blocking the insulin signal transduction in skeletal muscle via Jun N-terminal kinase. It was reported that traditional cigarettes cause dyslipidaemia, increase the inflammatory mediator and decrease insulin sensibility^7^. According to a large national study in China, the prevalence of diabetes in regular smokers is 15–30% higher than in nonsmokers^8^. There is little research on the effect of e-cigarettes on the body metabolism.

Based on the results of the current study, it was found that both traditional cigarettes and electronic cigarettes could affect blood levels of lipids and inflammatory factors. The dyslipidemia induced by smoking might be due to harmful substances in smoke stimulating the release of adrenaline from the adrenal cortex and so increasing the oxidative decomposition of adipose tissue. Therefore, the elevated free fatty acids stimulate the synthesis of triglycerides and low-density lipoproteins by hepatocytes and inhibit the synthesis of high-density lipoproteins by hepatic microsomes, which lead to dyslipidemia. Other studies have indicated that harmful substances in traditional smoke induce the activation and degranulation of mast cells in the respiratory tract, promoting the inflammation of airways^9-11^. Electronic cigarettes simulate the process of emission and inhaling of traditional cigarettes, using modern microelectronics technologies. Therefore, the combustion of cigarettes could be avoided with reduced emission of harmful substances such as tar, carbon monoxide etc.^12,13^ As a result, electronic cigarettes have been launched in the market as a substitute to traditional cigarettes. Although the composition of electronic liquid is relatively simple and there is no combustion process, a number of harmful components are produced in the process of atomization at high temperature, including acrolein, formaldehyde hemiacetals, and specific nitrosamines in tobacco^14,15^. In the current study, the effect of e-cigarettes without nicotine on the body metabolism was investigated in addition to e-cigarettes containing nicotine and traditional cigarettes. It was found that the levels of blood lipids and inflammatory factors were also significantly enhanced after exposure to e-cigarettes without nicotine. In the absence of nicotine, the physicochemical irritation of particles and alcohol compounds may affect the activity of repair proteins in lung tissue followed by damage to the respiratory system and induction of inflammation^16,17^. Hahn et al.^18^ determined the contents of major components in 54 electronic liquids and found that 1,2-propanediol and glycerol were the most abundant solvents. Although they are considered to be safe solvents via oral administration, some chemical reactions might be induced in the process of atomization of the electronic liquid. Propylene glycol could change into propylene oxide, which is classified as a B2B carcinogen by the International Cancer Research Institute. Glycerol could change to acrolein, which stimulates the upper respiratory tract and promotes inflammation^19,20^.

A number of studies have indicated that the enhanced level of TNF-α promotes the phosphorylation of serine and threonine, triggering the degradation insulin receptor substrates. The phosphorylation and the tyrosine kinase activity of insulin receptors is reduced, which induces the decrease of insulin sensibility^21,22^. According to the experiment of insulin tolerance, traditional cigarettes and electronic cigarettes with 12 mg/mL of nicotine significantly enhanced the insulin tolerance, whereas the electronic cigarette without nicotine showed no effect on the insulin tolerance compared to the fresh air control group. Other literature reported that nicotine and other alcohol compounds undergo complicated biological reactions in vivo and their metabolites cause DNA damage of repair proteins. Consequently, lesions on the respiratory tract and pulmonary functions cannot be efficiently repaired, resulting in repeated damage of the respiratory system^23-25^. The chronic toxic effect on the respiratory system by the generated metabolites would induce sustained and chronic damage to the respiratory system, causing damage of the vascular endothelial cells and increased coagulation of the blood vessels as well as reduced blood volume in skeletal muscles. Therefore, the insulin-mediated uptake of glucose is decreased, leading to reduced insulin sensibility^26-28^. Further experiments are planned to elucidate potential mechanisms.

## CONCLUSIONS

Traditional cigarettes and electronic cigarettes with/without nicotine increased the levels of blood lipids and chronic inflammation indexes and decreased the insulin sensibility in ApoE knockout mice, suggesting an effect of electronic cigarettes on body metabolism. The health hazards of electronic cigarettes should be carefully evaluated in order to supplement policies for public health.
